# Anti-Inflammatory Effects of 5α,8α-Epidioxycholest-6-en-3β-ol, a Steroidal Endoperoxide Isolated from *Aplysia depilans*, Based on Bioguided Fractionation and NMR Analysis

**DOI:** 10.3390/md17060330

**Published:** 2019-06-03

**Authors:** Renato B. Pereira, David M. Pereira, Carlos Jiménez, Jaime Rodríguez, Rosa M. Nieto, Romeu A. Videira, Olga Silva, Paula B. Andrade, Patrícia Valentão

**Affiliations:** 1 REQUIMTE/LAQV, Laboratório de Farmacognosia, Departamento de Química, Faculdade de Farmácia, Universidade do Porto, R. Jorge Viterbo Ferreira, nº 228, 4050-313 Porto, Portugal; ren.pereira@gmail.com (R.B.P.); ravideira@gmail.com (R.A.V.); pandrade@ff.up.pt (P.B.A.); 2 Departamento de Química, Facultade de Ciencias e Centro de Investigacións Científicas Avanzadas (CICA), Universidade da Coruña, E-15071 A Coruña, Spain; carlos.jimenez@udc.es (C.J.); jaime.rodriguez@udc.es (J.R.); rosa.nieto@udc.es (R.M.N.); 3 Research Institute for Medicines (iMed.ULisboa), Faculty of Pharmacy, Universidade de Lisboa, Av. Professor Gama Pinto, 1649-003 Lisbon, Portugal; osilva@campus.ul.pt

**Keywords:** *Aplysia depilans*, 5α,8α-epidioxycholest-6-en-3β-ol, iNOS, COX-1, COX-2, IL-6, TNF-α

## Abstract

Sea hares of *Aplysia* genus are recognized as a source of a diverse range of metabolites. 5α,8α-Endoperoxides belong to a group of oxidized sterols commonly found in marine organisms and display several bioactivities, including antimicrobial, anti-tumor, and immunomodulatory properties. Herein we report the isolation of 5α,8α-epidioxycholest-6-en-3β-ol (EnP(5,8)) from *Aplysia depilans* Gmelin, based on bioguided fractionation and nuclear magnetic resonance (NMR) analysis, as well as the first disclosure of its anti-inflammatory properties. EnP(5,8) revealed capacity to decrease cellular nitric oxide (NO) levels in RAW 264.7 macrophages treated with lipopolysaccharide (LPS) by downregulation of the *Nos2* (inducible nitric oxide synthase, iNOS) gene. Moreover, EnP(5,8) also inhibited the LPS-induced expression of cyclooxygenase-2 (COX-2), interleukin 6 (IL-6), and tumor necrosis factor alpha (TNF-α) at the mRNA and protein levels. Mild selective inhibition of COX-2 enzyme activity was also evidenced. Our findings provide evidence of EnP(5,8) as a potential lead drug molecule for the development of new anti-inflammatory agents.

## 1. Introduction

*Aplysia* sea hares are herbivorous mollusks that feed on a wide variety of green, red, and brown algae [1,2]. Indeed, it is worth mentioning the ability of these sea hares to use algae as a source of metabolites of their chemical defense system [1,3]. Moreover, *Aplysia* spp. complex digestive gland contributes to its metabolic machinery, leading to the production of innumerable compounds with interesting pharmacological properties [1,3,4]. Although the majority of secondary *Aplysia* metabolites have been extensively explored due to their cytotoxic and antibacterial properties, few reports address their anti-inflammatory activity.

During the last decades, marine organisms were shown to be a source of unconventional sterols. In particular, steroidal endoperoxides are a subgroup of steroids which are ubiquitously found in marine invertebrates, such as sponges, and sea hares, among others [5]. These marine sterols have been reported to possess diverse biological properties like anti-tumor, immunomodulatory, antiviral, and antifouling activities, being considered as lead drug compounds in the development of new pharmacological agents [6]. The 5α,8α-endoperoxides belong to a group of oxidized sterols derivatives resulting from the addition of an oxygen to a 5,7-diene system in the molecule of the precursor sterol [6]. Ergosterol peroxide is the best-known representative of this class, displaying anti-inflammatory properties that are associated to its capacity to inhibit the production of pro-inflammatory mediators and the activation of nuclear factor kappa B (NF-κB) signaling pathway [7]. The cholesterol derivative 5α,8α-epidioxycholest-6-en-3β-ol (EnP(5,8)) is another oxidized sterol that has been found in different species of sea hares [8,9], sea urchins, and cone snails [10,11,12]. Among the few studies addressing its biological activity, Minh et al. reported a strong cytotoxic effect against various cancer cell lines, namely human epidermoid carcinoma (KB), fibrillary sarcoma of uterus (FL), and human hepatocellular carcinoma (HepG-2) with IC_50_ values of 4.8, 9.4, and 5.8 µM, respectively [13]. More recently, Clark et al. found antileishmanial properties towards the amastigote form of *Leishmania donovani*, displaying an IC_50_ of 4.9 µM [14].

Herein, we report the isolation of EnP(5,8), based on bioguided fractionation and nuclear magnetic resonance (NMR) analysis, from a non-polar fraction of *Aplysia depilans* Gmelin methanolic extract, and the first disclosure of the molecular mechanisms underlying its anti-inflammatory properties in RAW 264.7 macrophages. Additionally, its capacity to inhibit 5-lipoxygenase (5-LOX), phospholipase A_2_ (PLA_2_), and cyclooxygenases (COX-1 and COX-2) was also explored.

## 2. Results and Discussion

### 2.1. Effect of Non-Polar Fraction of A. depilans Extract

#### 2.1.1. RAW 264.7 Macrophage Viability

**Figure 1:**
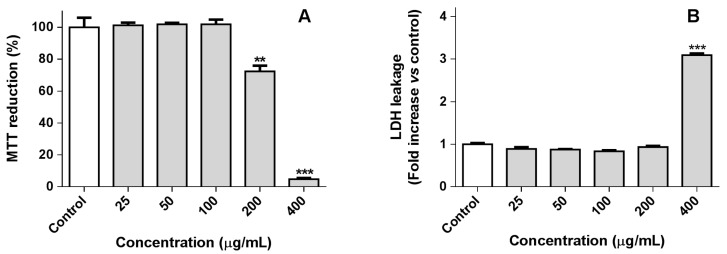
Effect of non-polar fraction of *Aplysia depilans* extract on 3-(4,5-dimethylthiazol-2-yl)-2,5-diphenyltetrazolium bromide (MTT) reduction (**A**) and lactate dehydrogenase (LDH) leakage (**B**) of RAW 264.7 macrophages. Results are expressed as mean ± standard error of the mean (SEM) of at least three independent experiments. ** *p* < 0.01, *** *p* < 0.001.

Continuing our ongoing research on the anti-inflammatory activity of *Aplysia* metabolites [1,4,15,16,17], this work gives particular attention to the lipophilic molecules. Prior to the assessment of the anti-inflammatory activity of the non-polar fraction of the *A. depilans* extract (see Section 3.3.), RAW 264.7 macrophages were incubated with five different concentrations of the extract (25–400 µg/mL) to find the non-cytotoxic concentrations. Macrophages viability was evaluated based on the reduction of 3-(4,5-dimethylthiazol-2-yl)-2,5-diphenyltetrazolium bromide (MTT) and lactate dehydrogenase (LDH) leakage assay, which are indicative of the mitochondrial activity and membrane integrity of the cells, respectively. As can be seen in Figure 1, the non-polar fraction of *A. depilans* extract was not cytotoxic at concentrations lower or equal to 100 µg/mL, which were used to perform subsequent experiments.

#### 2.1.2. Cellular Nitric Oxide Levels

**Figure 2:**
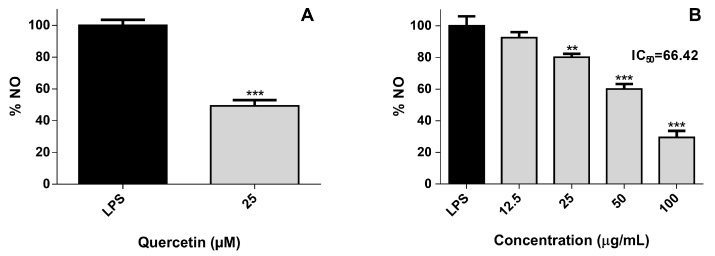
Effect in nitric oxide (NO) levels of cells pre-treated for 2 h with quercetin at 25 µM (**A**) and with the non-polar fraction of *A. depilans* extract (**B**), followed by 22 h co-treatment with 1 µg/mL of lipopolysaccharide (LPS). Results are expressed as mean ± SEM of at least three independent experiments. ** *p* < 0.01, *** *p* < 0.001.

Nitric oxide (NO) is an important signaling molecule synthesized by many cells in response to homeostatic and pathologic stimuli [18]. Although it was first described as a vasodilator, having a preponderant role in blood pressure, its association with the pathogenesis of several inflammatory conditions is fully established nowadays [18]. In the cell model used, a pro-inflammatory phenotype was induced using lipopolysaccharide (LPS), an endotoxin that triggers several inflammatory mediators, including NO. As can be seen in Figure 2, the pre-incubation of the non-polar fraction of *A. depilans* extract for 2 h leads to a decrease in cellular NO levels with an IC_50_ of 66.42 µg/mL. In order to find the compound(s) responsible for this effect, a bioguided fractionation of the above-mentioned fraction was performed, based on its capacity to reduce the cellular NO levels.

### 2.2. Effect of A. depilans Non-Polar Extract Sub-Fractions

Due to its promising anti-inflammatory activity, the non-polar fraction of *A. depilans* extract was fractionated, affording 11 fractions (Fr1–Fr11), according to the procedure described in Material and Methods (Section 3.4.). Then, as reported above for the crude extract, the effect of Fr1–Fr11 on MTT reduction was evaluated in RAW 264.7 macrophages (Figure S1A). Afterwards, the non-cytotoxic concentrations were screened for their ability to reduce the cellular NO levels, in order to find the most active fraction (Figure S1A). Despite the interesting activity demonstrated by Fr8, the IC_50_ could not be determined once this fraction was cytotoxic at concentrations higher or equal to 25 µg/mL. Fr6 was the fraction displaying the lowest determined IC_50_ value (25.60 µg/mL), significant differences being found for all tested concentrations (*p* < 0.05) (Figure S1A), which indicates the presence of active molecule(s). Hereupon, Fr6 was re-fractionated, leading to the isolation of the natural steroidal endoperoxide EnP(5,8) (Figure S1B, NMR data―Section 3.4 [12,13]). In order to evaluate the relevance of this compound to the anti-inflammatory properties of Fr6, the levels of NO after a pre-incubation with different concentrations of EnP(5,8) were measured.

### 2.3. Anti-Inflammatory Effect of EnP(5,8)

#### 2.3.1. RAW 264.7 Macrophages Viability

**Figure 3:**
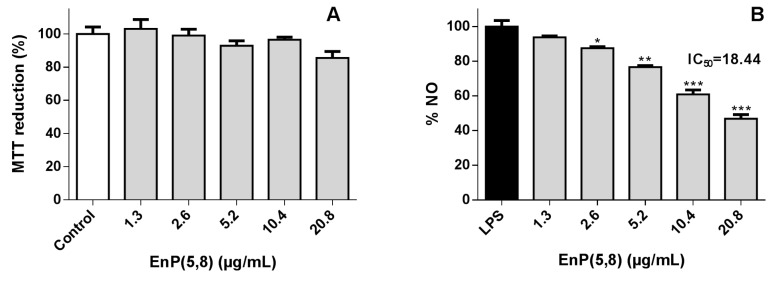
(**A**) Effect of 5α,8α-epidioxycholest-6-en-3β-ol (EnP(5,8)) on MTT reduction of RAW 264.7 macrophages. (**B**) Effect in NO levels of cells pre-treated for 2 h with EnP(5,8), followed by 22-h co-treatment with 1 µg/mL of LPS. Results are expressed as mean ± SEM of at least three independent experiments. * *p* < 0.05, ** *p* < 0.01, *** *p* < 0.001.

As can be seen in Figure 3A, the EnP(5,8) at 1.3–20.8 µg/mL (3.125–50 µM) had no significant effect on the cell viability (*p* > 0.05). Of note, in a promonocytic human cell line (THP-1), the structurally related ergosterol peroxide has demonstrated the capacity to dose-dependently (1–20 µM) increase the protection of cells against LPS-induced toxicity [7]. Relevantly, EnP(5,8) displayed an interesting anti-inflammatory effect: at 10.4 µg/mL (25 µM) the NO levels were reduced c.a. 40% in comparison with LPS (*p* < 0.001) (Figure 3B). Thus, our findings confirm the preliminary results reported by Huang et al. [19] on the ability of EnP(5,8) to reduce cellular NO levels. Of note, the same study carried out with some EnP(5,8) analogues evidenced that while the unsaturation at C22 does not appear to be a structural requirement for NO reduction, the substitution of C24 by an ethyl group apparently decreases its activity [19]. Multiple mechanisms of action can be involved in the observed effect: firstly, the EnP(5,8) can exert antioxidant activity, leading to a decrease of ^●^NO level; secondly, EnP(5,8) can inhibit inducible nitric oxide synthase (iNOS), triggering a reduction in NO and l-citrulline levels; and thirdly, the EnP(5,8) can modulate several pathways, leading to a decrease on iNOS mRNA and protein expression. In order to clarify the mechanism underlying its activity, EnP(5,8) was assessed for its capacity to scavenge ^●^NO and to modulate iNOS action.

#### 2.3.2. Nitric Oxide Radical (^●^NO) Scavenging Assay

Multiple antioxidant molecules have been associated to a decrease on NO production in LPS-stimulated macrophages, by a direct scavenging effect of ^●^NO [20]. Herewith and using a cell-free assay, the ^●^NO scavenging activity of EnP(5,8) was investigated, at the same concentrations tested for cellular NO (up to 20.8 µg/mL), which revealed no antioxidant effect (data not shown).

#### 2.3.3. iNOS Direct Inhibition

**Figure 4:**
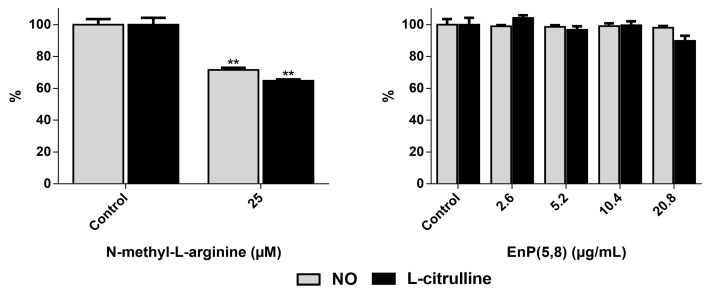
Effect of EnP(5,8) on the cellular levels of NO and l-citrulline, the two bio-products of inducible nitric oxide synthase (iNOS) activity. *N*-methyl-l-arginine was used as a positive control. The results correspond to the mean ± SEM of at least three independent experiments performed in duplicate. ** *p* < 0.01.

According to our method [15] and based on the conversion of l-arginine to equimolar amounts of NO and l-citrulline, the levels of these products can be used as markers of iNOS activity. Cells without LPS activation showed negligible activity, as expected (data not shown). As can be seen in Figure 4, the pre-incubation of the LPS-activated RAW 264.7 cells with the commercial inhibitor *N*-methyl-l-arginine significantly inhibited iNOS enzyme activity at 25 µM, decreasing to the same extent NO and l-citrulline levels (*p* < 0.01). On the other hand, no significant differences were found with the pre-incubation with EnP(5,8) (*p* > 0.05) (Figure 4), indicating that this molecule cannot directly inhibit iNOS enzyme activity. Once the reduction of the cellular NO levels reported in Figure 3B cannot be explained by a scavenging effect and/or to an enzyme inhibition process, the interference of EnP(5,8) with respect to the iNOS gene and protein expression was further studied.

#### 2.3.4. iNOS, COX-2, Interleukin 6 (IL-6), and Tumor Necrosis Factor Alpha (TNF-α) mRNA and Protein Expression

NF-κB has been shown to play an important role in regulating the expression of pro-inflammatory genes, including cytokines, chemokines, and adhesion molecules, which are involved in cell survival, immunity and inflammatory processes [21]. In RAW 264.7 macrophages, LPS is used to stimulate an inflammatory response, through NF-κB activation, leading to a marked increase in iNOS, COX-2, interleukin-6 (IL-6), and tumor necrosis factor alpha (TNF-α) expression [21]. As can be seen in Figure S2, the model is appropriate to study these events, as cells challenged with LPS expressed higher amounts of iNOS, COX-2, IL-6, and TNF-α mRNA, evidenced by the significant decrease observed in the number of amplification cycles (Ct values) (*p* < 0.001).

Since EnP(5,8) was found to decrease NO production, without any capacity to scavenge ^•^NO and/or to inhibit iNOS activity, we performed qPCR and western blotting to determine if the observed effects were related to iNOS expression. In addition and once LPS treatment also increased the mRNA and protein levels of COX-2, IL-6, and TNF-α, the effect of EnP(5,8) on them was also explored.

**Figure 5:**
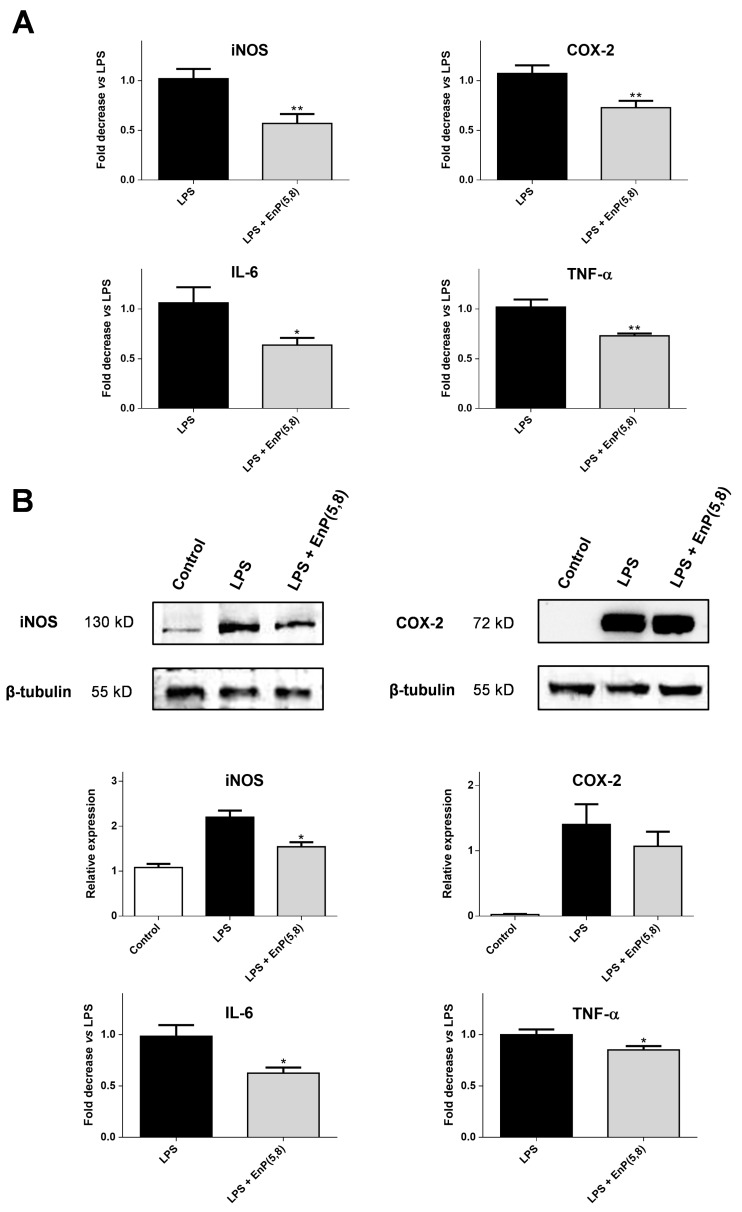
(**A**) Effect of EnP(5,8) on LPS-induced iNOS, cyclooxygenase-2 (COX-2), interleukin-6 (IL-6), and tumor necrosis factor alpha (TNF-α) mRNA expressions. RAW 264.7 cells were treated for 4 h with LPS (1 µg/mL), with or without pre-incubation with EnP(5,8) for 2 h. qPCR data were normalized to the reference gene, β-actin. (**B**) Effect of EnP(5,8) on LPS-induced iNOS, COX-2, IL-6, and TNF-α protein expression. RAW 264.7 cells were treated for 16 h with LPS (1 µg/mL), with or without pre-incubation with EnP(5,8) for 2 h. LPS: lipopolysaccharide; EnP(5,8): 5α,8α-epidioxycholest-6-en-3β-ol at 10.4 µg/mL (25 µM). Results are expressed as mean ± SEM of at least three independent experiments. * *p* < 0.05; ** *p* < 0.01.

The qPCR analysis showed that the expressions of iNOS, COX-2, IL-6 and TNF-α mRNA were significantly inhibited (43%, 28%, 38% and 15%, respectively) by EnP(5,8) at 10.4 µg/mL (25 µM) (*p* < 0.05) (Figure 5A). A similar effect was also reported with ergosterol peroxide, which demonstrated the ability to down-regulate mRNA expression of iNOS and COX-2 at 30 µM [22]. Moreover, western blotting analysis evidenced that iNOS protein levels were associated with their mRNA expression (Figure 5), suggesting that the observed reductions in NO production by EnP(5,8) were due to a transcriptional suppression of iNOS. Similarly, an apparent inhibition on iNOS protein expression was also noted by Lu et al. [23] upon treatment with an EnP(5,8) analogue, containing an extra methyl group on C24 of the side chain. Interestingly, a markedly increased activity was observed when the C24 methyl substitution was accompanied by cyclisation, arising a cyclopropane in the saturated side chain [23]. Concerning COX-2 protein expression, no significant inhibition was described for both EnP(5,8) analogues [23], which is in agreement with our findings (Figure 5B). Of note, the protein levels of COX-2, IL-6, and TNF-α were also associated with their mRNA expression (Figure 5). Curiously, a previous study involving different sterols and conducted in HEK293 cells transfected with an NF-κB-promoted luciferase reporter demonstrated the inhibitory capacity of 5α,8α-epidioxysterols on 12-O-tetradecanoylphorbol-13-acetate (TPA)-induced NF-κB luciferase activity [24]. The EnP(5,8) and its C22 unsaturated analogue proved this capacity, the latter being c.a. 3.2 fold more active [24]. Based on those findings, the authors suggested the 5α,8α-epidioxy as the active group and that the branched chain affects the potency of these molecules [24]. Herewith, the effects of EnP(5,8) observed on iNOS, COX-2, IL-6, and TNF-α mRNA and protein expression may be related to a down-regulation of this signaling pathway.

#### 2.3.5. Arachidonic Acid Pathway Enzymes

**Figure 6:**
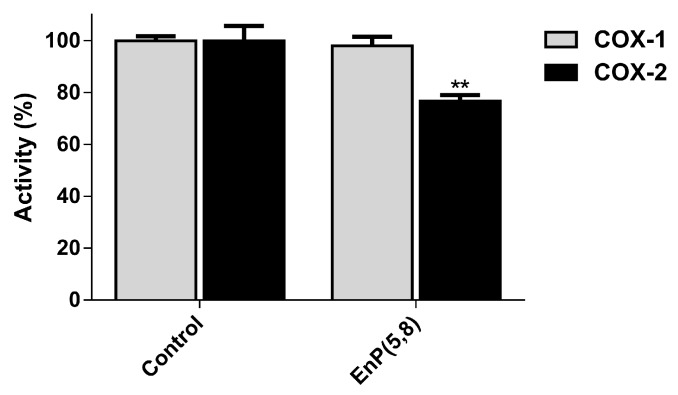
Effect of EnP(5,8) on COX-1 and COX-2 activity. EnP(5,8): 5α,8α-epidioxycholest-6-en-3β-ol at 20.8 µg/mL (50 µM). Results are presented as mean ± SEM of at least three experiments, performed in duplicate. ** *p* < 0.01 compared to the respective control.

There are several functionally distinct enzymes involved in the arachidonic acid (AA) pathway: phospholipase A_2_ (PLA_2_), cyclooxygenase (COX), and lipoxygenase (LOX). Under inflammatory conditions, AA is released through the action of PLA_2_ upon the membrane phospholipids, being further metabolized via several different enzymatic machineries, namely the LOX and COX pathways, giving rise to a group of pro-inflammatory mediators called eicosanoids [23]. The eicosanoid production is considerably increased during inflammation, with leukotrienes and prostaglandins being products of LOX and COX activity, respectively [25,26]. In regard to COX, there are distinct isoforms such as COX-1, which is constitutively expressed in a variety of cell types being involved in their homeostasis, and the inducible isoform, COX-2, with a specific tissue distribution, thus constituting the most relevant target of anti-inflammatory drugs [26,27,28]. Therefore, we evaluated the ability of EnP(5,8) to inhibit the above-mentioned enzymes, at non-cytotoxic concentrations. Concerning PLA_2_ and 5-LOX inhibition, EnP(5,8) demonstrated no measurable activity at concentrations up to 20.8 µg/mL (50 µM) (data not shown). Curiously, the structurally similar 5α,8α-epidioxy-(24S)-ergosta-6-en-3β-ol, differing by the presence of an additional methyl group in C24, proved also to be ineffective against *Apis mellifera* bee venom PLA_2_ [29]. Recently, a structure-activity relationship (SAR) study comparing the 5-LOX inhibitory capacities of some ergosterol derivatives demonstrated the importance of the peroxy ring for greater activity [30]. However, despite EnP(5,8) presenting the peroxy group, it does not contain the C22–C23 olefinic double bound, which has been revealed to be essential for 5-LOX inhibition, thus corroborating our findings [30]. On the other hand, despite no significant differences have been found up to 10.4 µg/mL (25 µM), EnP(5,8) at 20.8 µg/mL (50 µM) significantly inhibited (23%) COX-2 activity (*p* < 0.01), without notable inhibition of COX-1 (Figure 6). Even with its selectivity, the reported activity is weak when compared with celecoxib, a COX-2 inhibitor used in the clinic, which at the same concentration inhibits 74% of enzyme activity [31].

Noteworthy, a docking study carried out with sterols and triterpenoids proposed key structural requirements for COX binding, namely the presence of a tetracyclic skeleton, such as cholestane or stigmastane, the incorporation of an aliphatic chain in C17, and the presence of hydroxyl groups at C3 or C6, EnP(5,8) fulfilling these molecular features [32]. Despite the established structural requirements, few studies effectively evaluated the ability of this type of molecules to inhibit COX activity. Zhang, Mills and Nair (2003) screened the capacity of ergosterol and its peroxide, both containing the ergostane skeleton, on the inhibition of the two COX isoforms. They showed that the ergosterol peroxidation promotes a selectivity switch, leading to a higher COX-1 than COX-2 inhibitory activity [33]. In contrast, despite the presence of the peroxy ring in EnP(5,8), the selectivity to COX-2 was observed, suggesting that the substitution and unsaturation pattern of the C17 aliphatic chain, may also have a preponderant role in COX selectivity. As a matter of fact, Loza-Mejía and Salazar (2015) demonstrated that compounds with unsaturation in the aliphatic chain presented lower binding energies [32]. Overall, and due to the gastrointestinal tract side effects associated with the non-selective COX-2 inhibitors, EnP(5,8) may constitute an interesting molecule for the development of selective COX-2 inhibitors.

## 3. Materials and Methods

### 3.1. Reagents and Standards

LPS from *Escherichia coli*, sodium pyruvate, sulfanilamide, MTT, Triton X-100, *N*-(naphth-1-yl)ethylenediamine dihydrochloride, β-nicotinamide adenine dinucleotide reduced form (NADH), sodium deoxycholate, dimethyl sulfoxide (DMSO), trizma hydrochloride, trypan blue, soybean lipoxygenase (LOX) from *Glycine max* (L.) Merr. (Type V-S; EC 1.13.11.12), phospholipase A_2_ (PLA_2_) from honey bee (*Apis mellifera*) venom (EC.3.1.1.4), propan-2-ol, butan-1-ol, sodium nitroprusside (SNP), l-arginine monohydrochloride, diacetyl monoxime, antipyrine E, H_2_SO_4_, bovine serum albumin (BSA), chloroform, and *N*-methyl-l-arginine acetate salt were from Sigma-Aldrich (St. Louis, MO, USA). 1,2-Dilinoleoyl-sn-glycero-3-phosphocholine (DL-PC) was from Larodan (Solna, Sweden). Dulbecco’s Modified Eagle Medium (DMEM), fetal bovine serum (FBS), Hank’s balanced salt solution (HBSS) and Pen Strep solution (penicillin 5000 units/mL and streptomycin 5000 µg/mL) were purchased from GIBCO, Invitrogen (Grand Island, NE, USA). β-Tubulin primary antibody, as well as anti-rabbit secondary antibody, were from Santa Cruz Biotechnology (Dallas, TX, USA). PureZOL™ reagent was from Bio-Rad (Hercules, CA, USA). KAPA SYBR^®^ FAST qPCR Kit Master Mix (2X) Universal was from Kapa Biosystems (Boston, MA, USA). iNOS primary antibody was from Abcam (UK). COX-2 primary antibody, Qubit™ RNA HS assay kit, Qubit™ RNA IQ assay kit, SuperScript™ IV VILO™ Master Mix and primers were from Invitrogen by Thermo Fisher Scientific (Waltham, MA, USA). WesternBright ECL HRP substrate was from Advansta (Menlo Park, CA, USA). Potassium dihydrogen phosphate was purchased from Merck (Darmstadt, Germany). Methanol was from Chem-Lab (Zedelgem, Belgium) and hexane from Fisher Chemical (Loughborough, UK). Mouse IL-6 ELISA MAX™ Deluxe and Mouse TNF-α ELISA MAX™ Deluxe were purchased from BioLegend Inc. (San Diego, CA, USA). COX fluorescent inhibitor screening assay kit was from Cayman chemical (Ann Arbor, MI, USA).

### 3.2. Sample

*A. depilans* specimens were collected by hand at Praia da Memória (GPS coordinates 41°13’59.0”N, 8°43’28.1”W), north Portugal, at a depth of 1–3 feet, in June 2015. Specimens were immediately placed on ice and transported to the laboratory. The opisthobranch mollusks were then washed with sea water and kept at −20 °C, until use. Species identification was performed by Prof. Alexandre Lobo-da-Cunha (Centre of Marine and Environmental Research (CIIMAR), Porto, Portugal).

### 3.3. Extraction

*A. depilans* specimens (5.55 kg, wet weight) were ground using an Ultra-Turrax homogenizer. The resulting biomass was exhaustively extracted with methanol, at room temperature. Afterwards, the extracting solution was filtered using a Büchner funnel and concentrated under reduced pressure. The remaining aqueous layer, derived from the water contained in the mollusks, was partitioned with hexane [34] and evaporated to dryness to give a non-polar fraction containing 10.07 g of organic residue.

### 3.4. Extract Fractionation

A part of the non-polar fraction of *A. depilans* extract (2.0 g) was submitted to a flash chromatography on a silica gel column (90 g, 5.0 cm × 20 cm), using a gradient mixture of Hexane-EtOAc with increasing polarity, to afford eleven fractions (Fr1–Fr11). Fr6 (226.5 mg), eluted with 50% of hexane in EtOAc, was further fractionated by flash chromatography on a silica gel column (17 g, 2.0 cm × 20 cm), eluting with an isocratic mixture of hexane:EtOAc (40:60), to give nine sub-fractions (subFr1–subFr9). In order to exclude redundant compounds and/or chemical nullities, ^1^H-NMR-based dereplication of the sub-fractions allowed the detection of cholesterol and mixtures of common glycerolipids, containing some unsaturated fatty acids, which are extremely explored and recognized for their anti-inflammatory properties. The steroidal endoperoxide was detected in subFr5 and subFr7, and since there are no studies addressing its anti-inflammatory effects, these sub-fractions were selected. SubFr5 (11.9 mg) was separated by HPLC (Agilent HP 1100) (see chromatogram in Figure S3A), using a Symmetry 300™ column, 3.5 µm, 4.6 mm × 150 mm, IR detector (Agilent) with an isocratic mobile phase of MeOH:H_2_O (98:2) and a flow of 1 mL/min to afford EnP(5,8) (*t*_R_ = 4.75 min, 0.6 mg). SubFr7 (22.2 mg) was separated by HPLC (Agilent HP 1100) (see chromatogram in Figure S4), using a Prep Nova-Pak HR Sílica column, 60 Å, 6 µm, 3.9 mm × 300 mm, UV detector at 280 nm (Agilent), with an isocratic mobile phase of Hexane:EtOAc (80:20) and a flow of 1 mL/min to afford EnP(5,8) (*t*_R_ = 15.08 min, 3.3 mg). Total yield of EnP(5,8) is 3.54 × 10^−4^% w/w.

^1^H-NMR (500 MHz, CDCl_3_) δ: 6.51 (d, 8.5 Hz, H7); 6.24 (d, 8.5 Hz, H6); 3.96 (m, H3); 2.11/1.90 (m, H4); 1.94/1.68 (m, H1); 1.83/1.52 (m, H2); 1.50/1.21 (m, H15); 1.49 (m, H25); 1.47 (m, H9); 1.21 (m, H12); 0.90 (3H, d, 6.5 Hz, H21); 0.88 (3H, s, H19); 0.86 (3H, d, 6.5 Hz, H26), 0.86 (3H, d, 6.5 Hz, H27); 0.80 (3H, s, H18) (See ^1^H-NMR spectrum in Figure S5A).

^13^C-NMR (125 MHz, CDCl_3_) δ: 135.4 (CH, C6); 130.8 (CH, C7); 82.1 (C, C5); 79.4 (C, C8); 66.5 (CH, C3); 56.4 (CH, C17); 51.6 (CH, C14); 51.0 (CH, C9); 44.7 (C, C13); 39.4 (CH_2_, C12, C24); 36.9 (C, C10); 36.9 (CH_2_, C4); 35.9 (CH_2_, C22); 35.2 (CH, C20); 34.7 (CH_2_, C1); 30.1 (CH_2_, C2); 28.2 (CH_2_, C16); 28.0 (CH, C25); 23.8 (CH_2_, C23); 23.4 (CH_2_, C11); 22.8 (CH_3_, C27); 22.5 (CH_3_, C26); 20.6 (CH_2_, C15); 18.6 (CH_3_, C21); 18.2 (CH_3_, C19); 12.6 (CH_3_, C18) (See ^13^C-NMR spectrum in Figure S5B).

EIMS (70 ev): *m/z* (rel. int.) 416, (M^+^, 5); 398, (7); 384, (100), 334, (80).

### 3.5. Cell Culture

RAW 264.7 macrophages were provided by American Type Culture Collection (LGC Standards S.L.U., Barcelona, Spain). Cells were cultured in DMEM supplemented with 10% FBS and 1% penicillin/streptomycin, and were incubated at 37 °C, in a humidified atmosphere of 5% CO_2_.

### 3.6. MTT Reduction Assay

RAW 264.7 cells were seeded in 96-well plates at a density of 25,000 cells/well. After 24 h of growth, different concentrations of *A. depilans* extract/fractions/EnP(5,8) were added and plates were incubated for 24 h. Afterwards, mitochondrial activity was evaluated by the ability of metabolically active cells to convert yellow tetrazolium MTT to formazan. Results are expressed as percentage of the respective control and correspond to the mean ± SEM of at least three independent experiments performed in triplicate.

### 3.7. Membrane Integrity Assay

LDH release into the media is usually used as a marker for loss of membrane integrity. To assess the release of this cytosolic enzyme, a protocol described by Silva et al. was followed [35]. Briefly, 24 h after the incubation of the cells with *A. depilans* extract/fractions/EnP(5,8), 20 µL of culture media were removed to a 96-well plate. Then, 25 µL of pyruvate was added to each well, followed by 230 µL of NADH. LDH released was evaluated at 340 nm, in a Multiskan GO plate reader (Thermo Fisher Scientific; Waltham, MA, USA), by monitoring the oxidation of NADH during the conversion of pyruvate to lactate. 1% Triton X-100 was used as positive control to assure cell lysis (30 min). All the results correspond to the fold-increase of absorbance in treated vs. untreated cells of, at least, three independent experiments performed in triplicate.

### 3.8. Determination of NO Levels

RAW 264.7 cells were cultured in 96-well plates (35 000 cells/well) and incubated for 24 h at 37 °C and 5% CO_2_. Then, the medium of each well was replaced by different concentrations of *A. depilans* extract/fractions/EnP(5,8) or media only, followed by a stimulation with 1 µg/mL of LPS 2 h later. After 22 h challenge with LPS, the quantity of NO in cell culture medium was measured according to a previously described method [16]. Briefly, the nitrite resulting from the conversion of NO in culture medium was quantified mixing 75 µL of culture media with an equal volume of Griess reagent in a 96-well plate. The plate was incubated for 10 min, in the dark, and the absorbance was measured at 560 nm in a Multiskan GO plate reader (Thermo Fisher Scientific; Waltham, MA, USA). Quercetin was used as positive control. The results correspond to the mean ± SEM of at least three independent experiments performed in triplicate and are expressed as % NO in cells exposed to LPS (positive control for NO production).

### 3.9. ^●^NO Scavenging Assay

^●^NO generated from SNP was measured according to a previously described method [15]. Briefly, the reaction mixture, containing 75 µL of SNP in phosphate buffer, with or without 75 µL of different concentrations of EnP(5,8) was incubated at room temperature for 1 h, under light. The ^●^NO scavenging capacity was determined adding 75 µL of Griess reagent and the absorbance was read at 560 nm after 10 min incubation in the dark. Three independent experiments were performed in triplicate.

### 3.10. iNOS Inhibition Assay

iNOS activity was evaluated based on the measurement of the two bio-products, NO and l-citrulline, after its action on l-arginine. RAW 264.7 cells (90000 cells/well) were seeded in 48-well plates and treated for 22 h with LPS (1 µg/mL). After this period of activation, which lead to an increased expression of iNOS enzyme, the culture medium was removed and cells were washed with HBSS and pre-incubated with different concentrations of EnP(5,8) in HBSS (2.6–20.8 µg/mL; 6.25–50 µM) for 1 h. Simultaneously, *N*-methyl-l-arginine (commercial iNOS inhibitor) was used as positive control. Then, l-arginine was added to each well, at a final concentration of 50 µM, and allowed to react during 2 h. Afterwards, the levels of NO and l-citrulline were determined using the Griess reagent and a previously described method [15]. The results correspond to the mean ± SEM of at least three independent experiments performed in duplicate.

### 3.11. RNA Extraction, Quantification, Integrity, and Conversion

RAW 264.7 cells (500000 cells/well) were seeded in 6-well plates and treated for 4 h with LPS (1 µg/mL), with or without pre-incubation with EnP(5,8) for 2 h. Afterwards, the supernatant was removed and the cells were disrupted in 1 mL of PureZOL™ reagent. Then, the samples were transferred to a RNase-free tube and 0.2 mL of chloroform were added. The mixture was shaken vigorously for 15 s. After 5 min incubation at room temperature, the samples were centrifuged at 12,000× *g* for 15 min at 4 °C. Following centrifugation, the aqueous phase containing the RNA was immediately transferred to a new RNase-free tube and 0.5 mL of isopropyl alcohol were added, and mixture was incubated at room temperature for 5 min. Afterwards, the tubes were centrifuged at 12,000× *g* for 10 min at 4 °C, the RNA appearing as a white pellet on the side and bottom of the tube. Supernatant was carefully discarded and the RNA pellet was washed with 1 mL of 75% of ethanol. After vortexing, the mixture was centrifuged at 7500× *g* for 5 min at 4 °C and the supernatant was carefully discarded. Then, the RNA pellet was air-dried for about 5 min and reconstituted in 50 µL of PCR grade water. Subsequently, the RNA was quantified in a Qubit 4 fluorometer (Invitrogen by Thermo Fisher Scientific; Waltham, MA, USA), using the Qubit™ RNA HS assay kit. The RNA quality and integrity was then evaluated using the Qubit^TM^ RNA IQ assay kit. In order to obtain the complementary DNA, 1 µg of RNA was mixed with 4 µL SuperScript™ IV VILO™ Master Mix in a 20-µL reaction. The reverse-transcribed reaction involved three steps: incubation at 25 °C for 10 min, incubation at 50 °C for 10 min, and incubation at 85 °C for 5 min.

### 3.12. qPCR Analysis

qPCR analysis were conducted on multiple genes, namely iNOS, COX-2, IL-6, and TNF-α (Table 1). β-Actin was used as reference gene (Table 1). The primers were designed using the Primer-BLAST tool (NCBI, Bethesda, MD, USA) and synthesized by Thermo Fisher (Waltham, MA, USA), as listed in Table 1.

Real-time qPCR was performed using KAPA SYBR^®^ FAST qPCR Kit Master Mix (2X) Universal. The thermal cycling conditions were as follows: 3 min at 95 °C, followed by 40 cycles of denaturation at 95 °C for 3 s, specific annealing temperatures for each gene (Table 1) for 20 s, and extension at 72 °C for 20 s. The fluorescence signal was detected at the end of each cycle. The results were analyzed with qPCRsoft 4.0 supplied with the equipment qTOWER3 G (Analytik Jena AG, Germany), and a melting curve was used to confirm the specificity of the products. The expression levels of the target genes were normalized to the reference gene β-actin. At least five independent experiments were performed and all reactions were done in duplicate to confirm reproducibility. Results correspond to the mean ± SEM and are expressed as fold decrease vs. LPS.

### 3.13. Western Blot Analysis

Western blot analysis was performed according to a previously described method [36]. Briefly, RAW 264.7 cells (500000 cells/well) were seeded in 6-well plates and treated for 16 h with LPS (1 µg/mL), with or without pre-incubation with EnP(5,8) for 2 h. Afterwards, cells were washed with HBSS and lysed on ice, for 20 min with RIPA buffer, containing 1% of protease inhibitor cocktail. Then, cell debris were removed by microcentrifugation (14,000× *g* for 15 min), and supernatants were frozen at −80 °C. BSA was used as reference in order to quantify the total protein in cell lysates by the Bradford method [37]. Proteins (40 μg) were denatured at 70 °C, separated on 10% sodium dodecylsulfate (SDS)-polyacrylamide minigels and transferred onto nitrocellulose membranes, using a Trans-Blot^®^ Turbo (Bio-Rad; Hercules, CA, USA). Membranes were blocked for 1 h at room temperature with a solution of 5% skimmed milk powder in PBS 0.1% Triton X-100 and subsequently incubated overnight at 4 °C with specific antibodies against iNOS (1:200), COX-2 (1:2000) and β-tubulin (1:200). After washing, membranes were incubated with secondary antibody (1:1750) at room temperature for 1 h. Immunoreactive bands were visualized by adding WesternBright ECL HRP substrate and detecting the luminescent signal in a ChemiDoc™ Imaging System (Bio-Rad, Hercules, CA, USA). The relative optical density of bands was quantified by densitometry and normalized with respect to β-tubulin (loading control). The results correspond to the mean ± SEM of three independent experiments.

### 3.14. Enzyme-Linked Immunosorbent Assay (ELISA)

RAW 264.7 macrophages were seeded and treated for 16 h with LPS (1 µg/mL), with or without pre-incubation with EnP(5,8), as described in Section 3.13. The concentrations of the pro-inflammatory cytokines IL-6 and TNF-α were determined in the supernatants using an ELISA kit, according to the manufacturer’s instructions (BioLegend Inc.; San Diego, CA, USA). The concentration of each cytokine in the cell culture medium was normalized to total protein content, measured by Bradford method [28] using a BSA calibration curve as reference. Results correspond to the mean ± SEM of at least four independent experiments and are expressed as fold decrease vs. LPS.

### 3.15. PLA_2_ Inhibition Assay

The PLA_2_ inhibition assay was based on an indirect enzymatic method, which uses 5-LOX as coupling enzyme [38]. Briefly, 20 µL of PLA_2_ (1.75 µg/mL) and 20 µL of 5-LOX (1.61 µg/mL) in 3 mM deoxycholate dissolved in 50 mM Tris-HCL buffer (pH 8.5) were mixed with 50 µL of different concentrations of EnP(5,8) dissolved in the same buffer (3.125–50 µM). The reaction was initiated with the addition of 20 µL of DL-PC (PLA_2_ substrate) at 455 µM in 10 mM deoxycholate dissolved in 50 mM Tris-HCL buffer (pH 8.5). Then, the linoleic acid released through PLA_2_ activity was oxidized by 5-LOX at 37 °C, the increase in absorbance at 234 nm being followed spectrophotometrically in a Multiskan GO plate reader (Thermo Fisher Scientific; Waltham, MA, USA). Three independent experiments were performed in triplicate.

### 3.16. 5-LOX Inhibition Assay

The capacity of EnP(5,8) to inhibit 5-lipoxygenase was assessed according to a previously described method [39]. Briefly, 20 µL of EnP(5,8), 20 µL of soybean lipoxygenase (100 U) (Sigma-Aldrich; St. Louis, MO, USA) and 200 µL of phosphate buffer (pH 9) were pre-incubated at room temperature, for 5 min, in a 96-well plate. Then, 20 µL of linoleic acid (4.18 mM in ethanol) were added and the absorbance was monitored during 3 min, at 234 nm, using a Multiskan GO plate reader (Thermo Fisher Scientific; Waltham, MA, USA). Three independent experiments were performed in triplicate.

### 3.17. COX-1 and COX-2 Inhibition Assay

The assay was performed using the COX fluorescent inhibitor screening assay kit (Cayman chemical; Ann Arbor, MI, USA), with some modifications. It utilizes the peroxidase component of COXs and is based on the reaction between prostaglandin G_2_ (PGG_2_) (product of COX activity) and 10-acetyl-3,7-dihydroxyphenoxazine (ADHP), which produces a highly fluorescent compound, resorufin. Briefly, 60 µL of assay buffer (100 mM Tris-HCl, pH 8.0), 5 µL of hemin, 5 µL of enzyme (either COX-1 or COX-2), and 5 µL of EnP(5,8) were added in a black 96-well plate. After 5 min of incubation at room temperature, 5 µL of ADHP and 20 µL of a solution containing arachidonic acid (0.5 mM) and KOH (2.5 mM) were added to each well. After 2 min at room temperature, the fluorescence of resorufin was monitored with an excitation wavelength between 530–540 nm and an emission wavelength between 585–595 nm. SC-560 and DuP-697 inhibitors were used as positive controls to COX-1 and COX-2 assay, respectively. The results correspond to the mean ± SEM of at least three independent experiments performed in duplicate.

### 3.18. Statistical Analysis

Data analysis was performed using GraphPad Prism 6.01 Software (San Diego, CA, USA). Grubbs’ test was used to detect and exclude outliers. Prior to the analysis, the data set was checked for normality of distribution using the Shapiro–Wilk test, ensuring that all data followed a normal distribution. Levels of significance were determined using unpaired Student’s *t*-test where all columns of treatments were compared to the control. All data were expressed as mean ± SEM. Values of *p* < 0.05 were considered statistically significant.

## 4. Conclusions

In this work we evaluated the anti-inflammatory activity of a purified extract of *A. depilans*, and performed the bioguided isolation of the steroidal endoperoxide, EnP(5,8). In addition to the purified extract, the EnP(5,8) isolated from the most active sub-fraction demonstrated the ability to decrease the cellular NO levels in LPS-stimulated RAW 264.7 macrophages. Mechanistic studies evidenced that this decrease was due to a reduction in iNOS mRNA and protein expression. Moreover, EnP(5,8) also decreased the mRNA and protein expression of other inflammation mediators involved in the NF-κB pathway, namely COX-2, IL-6, and TNF-α. Even with a non-significant decrease in COX-2 protein levels, the overall results suggest that EnP(5,8) may act by an inhibitory effect upon the NF-κB activation. Furthermore, enzymatic assays carried out with multiples enzymes involved in the AA pathway showed a selective inhibition of COX-2 by the same molecule. The above-mentioned findings provided further clues on the potential of EnP(5,8) as an anti-inflammatory agent.

## Figures and Tables

**Table 1:** qPCR target information.

Gene	Accession Number	Primers	Annealing Temperature (°C)	Amplicon Length (bp)
*Nos2* (iNOS)	NM_001313921.1	F: CCGCCGCTCTAATACTTA R: TTCATCAAGGAATTATACA	58.0	121
*Ptgs2* (COX-2)	NM_011198.4	F: TGAGTACCGCAAACGCTTCT R: CAGCCATTTCCTTCTCTCCTGT	60.0	74
*Il6* (IL-6)	NM_001314054.1	F: AGACAAAGCCAGAGTCCTTCAG R: TGACTCCAGCTTATCTCTTGGT	59.0	75
*Tnf* (TNF-α)	NM_001278601.1	F: ACTGAACTTCGGGGTGATCG R: GTGGTTTGTGAGTGTGAGGGT	59.0	100
*Actb* (β-actin)	NM_007393.5	F: TATAAAACCCGGCGGCGCA R: TCATCCATGGCGAACTGGTG	61.5	117

## Supplementary Materials

The following are available online at https://www.mdpi.com/1660-3397/17/6/330/s1, Figure S1. (A) Effect of the *A. depilans* non-polar extract fractions on MTT reduction and in NO levels of RAW 264.7 macrophages challenged with 1 µg/mL of LPS. Results are expressed as mean ± SEM of at least three independent experiments. * *p* < 0.05, ** *p* < 0.01, *** *p* < 0.001. (B) Chemical structure of EnP(5,8) isolated from the non-polar fraction of *A. depilans* extract. Figure S2. Number of cycles (Ct values) required to amplify 2 ng of cDNA obtained from mRNA extracted from non-treated (control) and treated (LPS) RAW 264.7 macrophages. β-actin was used as reference gene. Results represent the mean ± SEM of at least five independent experiments performed in duplicate. *** *p* < 0.001 (vs. control). Figure S3. (A) HPLC chromatogram of SubFr5. (B) ^1^H-NMR (500 MHz) spectra in CDCl_3_ of the peak eluted at 4.75 min. Figure S4. HPLC chromatogram of SubFr7. Figure S5. (A) ^1^H-NMR (500 MHz), and (B) ^13^C-NMR (125 MHz) spectra in CDCl_3_ of the peak eluted at 15.08 min of Figure S4.
