# Supplementary material for: Anti-Inflammatory Effects of 5α,8α-Epidioxycholest-6-en-3β-ol, a Steroidal Endoperoxide Isolated from Aplysia depilans, Based on Bioguided Fractionation and NMR Analysis
Source: Mar Drugs. 2019 Jun 3;17(6):330. doi: 10.3390/md17060330 (PMC6628248; doi:10.3390/md17060330)
Supplement: Supplementary file 1 [file marinedrugs-17-00330-s001.pdf]

Supplementary Information

Anti-Inflammatory Effects of 5α,8α-Epidioxycholest-6-en-3β-ol, a Steroidal Endoperoxide Isolated from *Aplysia depilans*, Based on Bioguided Fractionation and NMR Analysis

Renato B. Pereira [1], David M. Pereira [1,*], Carlos Jiménez [2], Jaime Rodríguez [2], Rosa M. Nieto [2], Romeu A. Videira [1], Olga Silva [3], Paula B. Andrade [1] and Patrícia Valentão [1,*]

[1] REQUIMTE/LAQV, Laboratório de Farmacognosia, Departamento de Química, Faculdade de Farmácia, Universidade do Porto, R. Jorge Viterbo Ferreira, nº 228, 4050-313 Porto, Portugal; ren.pereira@gmail.com (R.B.P.); ravideira@gmail.com (R.A.V.); pandrade@ff.up.pt (P.B.A.)

[2] Departamento de Química, Facultade de Ciencias e Centro de Investigacións Científicas Avanzadas (CICA), Universidade da Coruña, A Coruña E-15071, Spain; carlos.jimenez@udc.es (C.J.); jaime.rodriguez@udc.es (J.R.); rosa.nieto@udc.es (R.M.N.)

[3] Research Institute for Medicines (iMed.ULisboa), Faculty of Pharmacy, Universidade de Lisboa, Av. Professor Gama Pinto, 1649-003, Lisbon, Portugal; osilva@campus.ul.pt

* Correspondence: dpereira@ff.up.pt (D.M.P.); valentao@ff.up.pt (P.V.); Tel.: +35-122-042-8653 (P.V.); Fax: +35-122-609-3390 (P.V.)

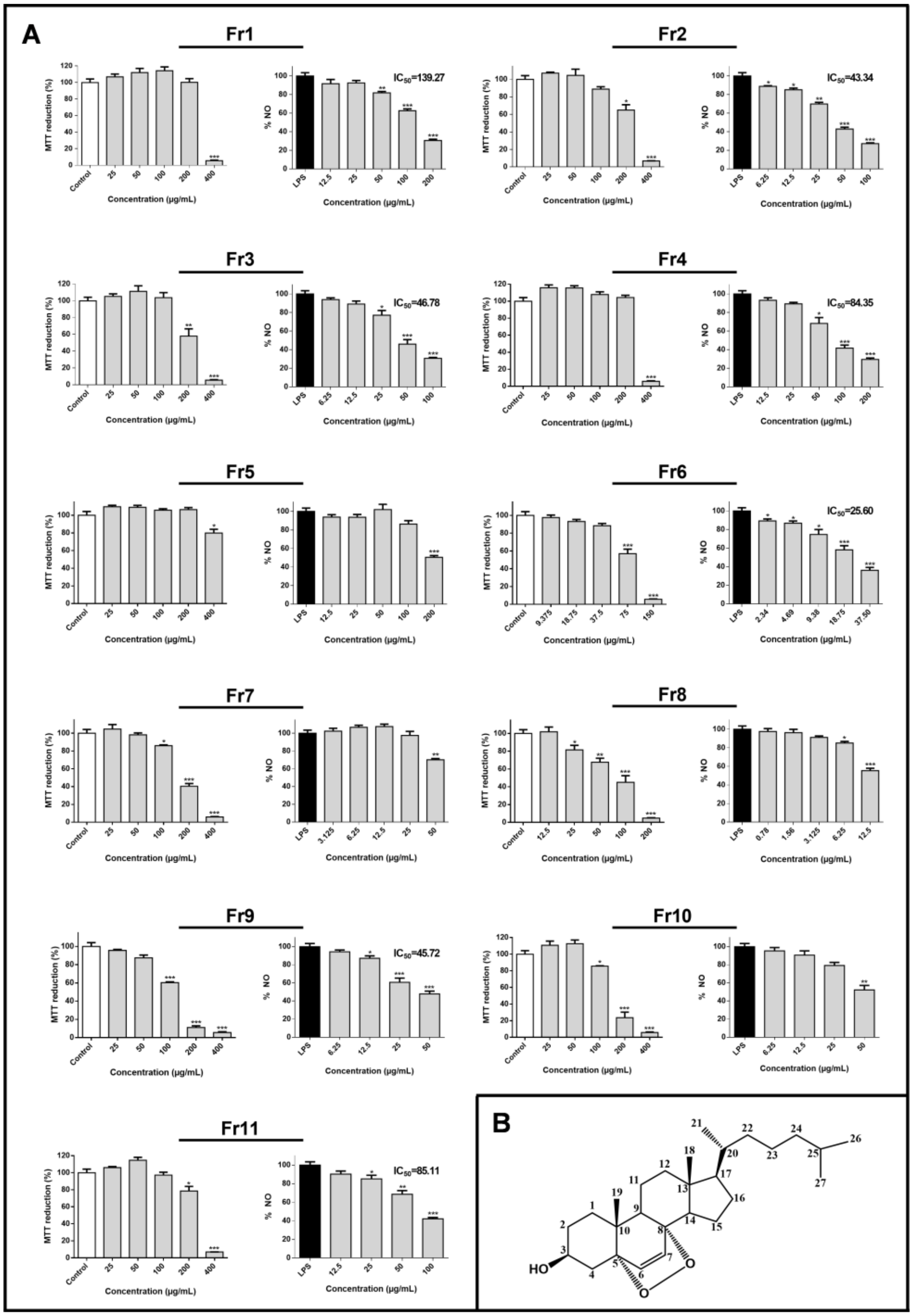

Figure S1. (A) Effect of the *A. depilans* non-polar extract fractions on MTT reduction and in NO levels of RAW 264.7 macrophages challenged with 1 µg/mL of LPS. Results are expressed as mean ± SEM of at least three independent experiments. *p<0.05, **p<0.01, ***p<0.001. (B) Chemical structure of EnP(5,8) isolated from the non-polar fraction of *A. depilans* extract.

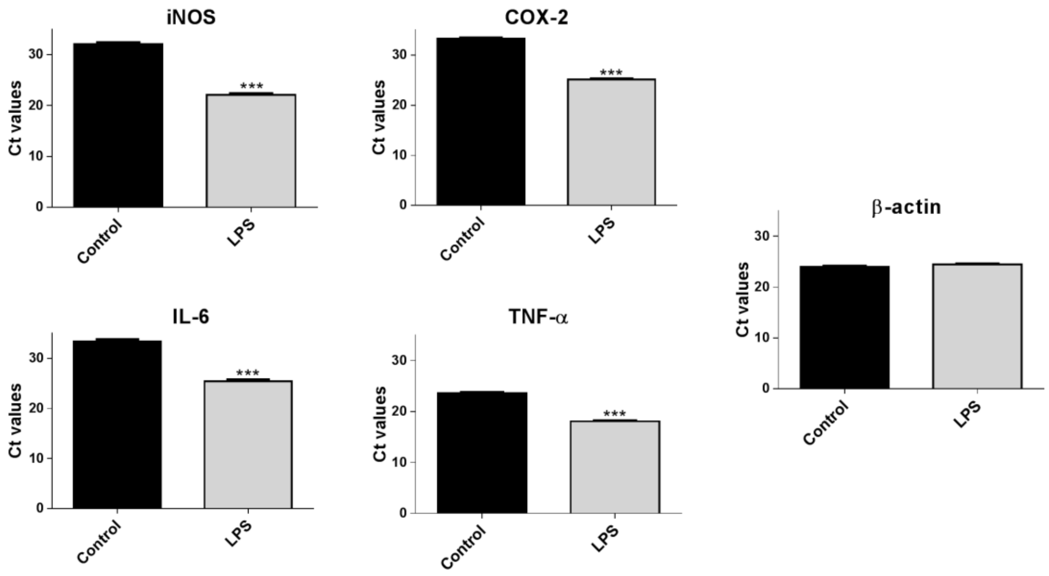

Figure S2. Number of cycles (Ct values) required to amplify 2 ng of cDNA obtained from mRNA extracted from non-treated (control) and treated (LPS) RAW 264.7 macrophages. β-actin was used as reference gene. Results represent the mean ± SEM of at least five independent experiments performed in duplicate. *** p <0.001 (vs. control).

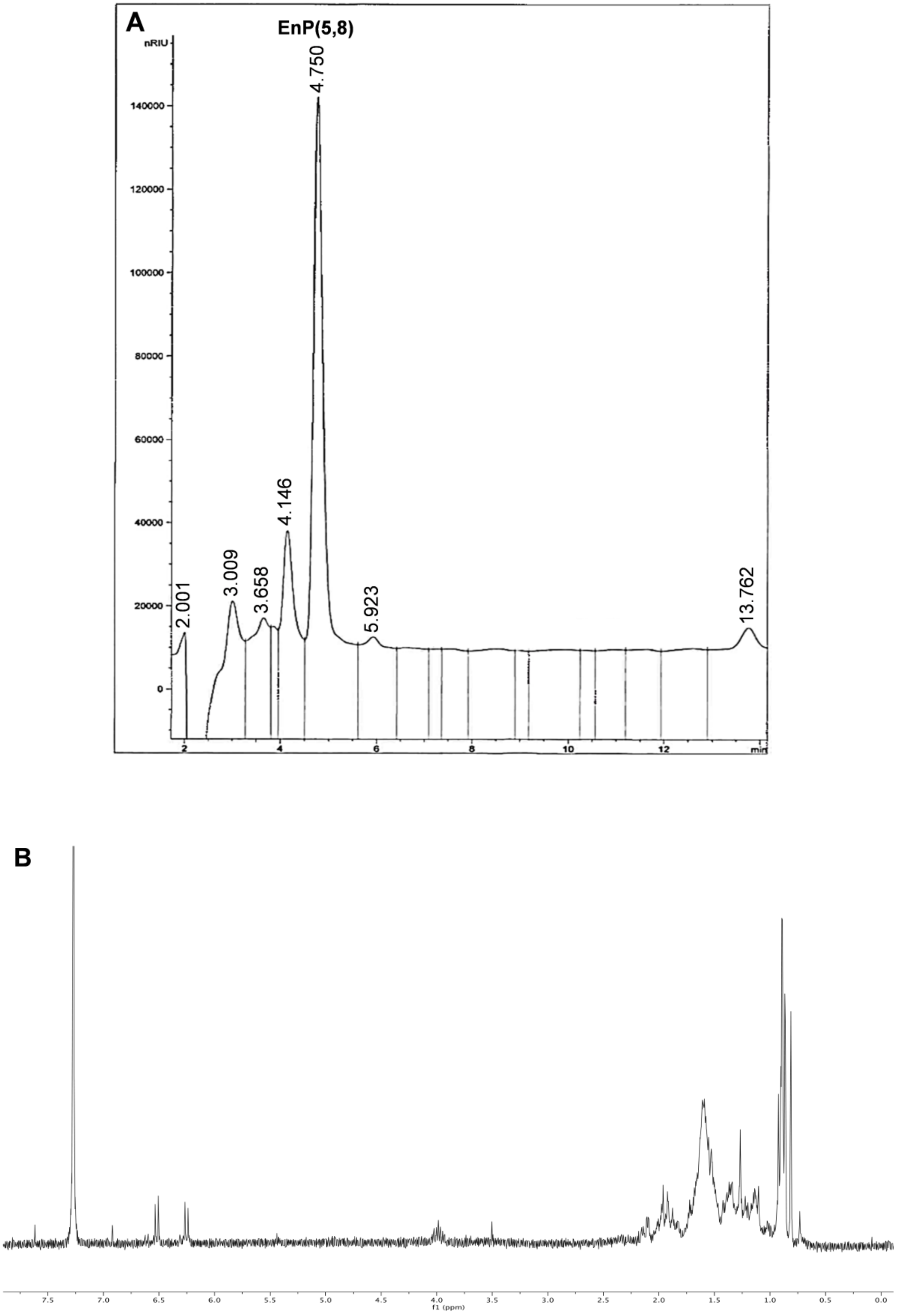

Figure S3: (A) HPLC chromatogram of SubFr5. (B) ^{1}H-NMR (500 MHz) spectra in $CDCl_3$ of the peak eluted at 4.75 min.

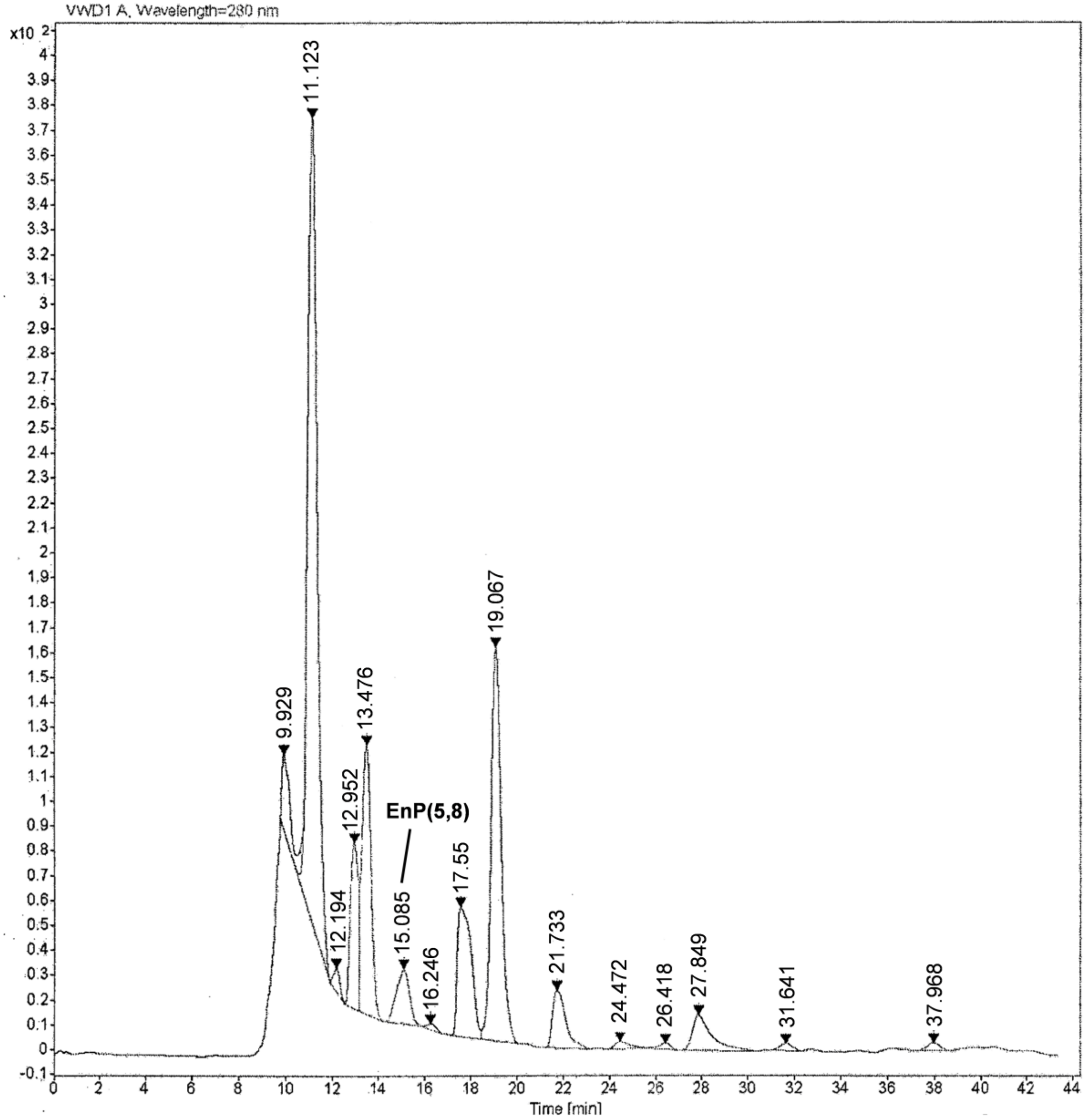

Figure S4. HPLC chromatogram of SubFr7.

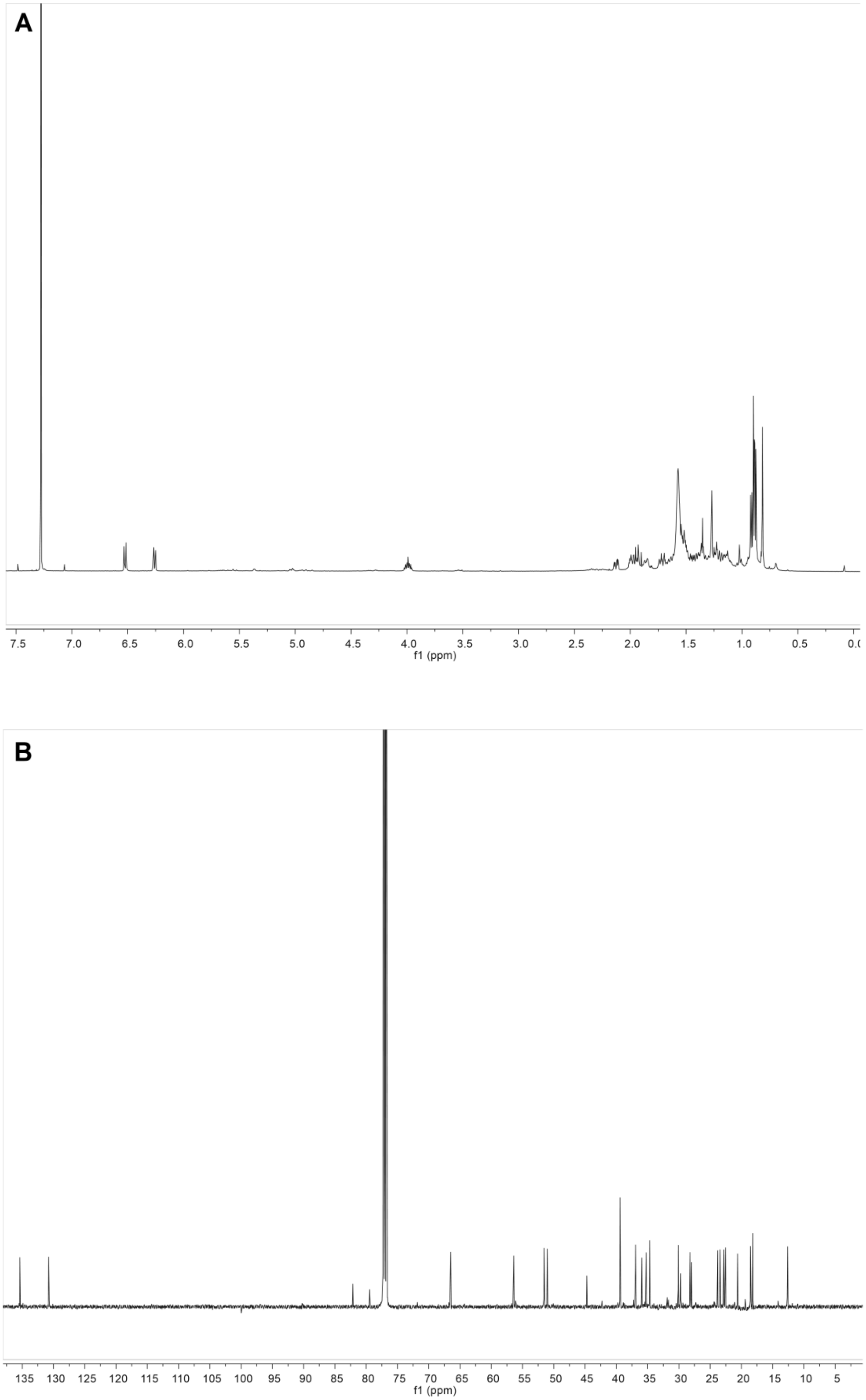

Figure S5. (A) ^{1}H-NMR (500 MHz), and (B) ^{13}C-NMR (125 MHz) spectra in $CDCl_3$ of the peak eluted at 15.08 min of Figure S4.
